# Evaluation of antimicrobial activity of psidium guajava species

**DOI:** 10.1186/1753-6561-8-S4-P79

**Published:** 2014-10-01

**Authors:** Thaís Honório Lins, Regina Célia Sales Santos Veríssimo, Raissa Fernanda Evangelista Santos, Maria Lysete de Assis Bastos, Rogério César Correia Bernardo, Valter Alvino, Patrícia de Albuquerque Sarmento, João Xavier Araújo Júnior

**Affiliations:** 1 Federal University of Alagoas, Maceió, Brazil

## Background

The *Psidium guajava*, commonly known as the apple guava, has shown various biological activities related to its main constituents, such as flavonoids, carotenoids, terpenoids and phenolic compounds. Studies emphasize anti-spasmodic and antimicrobial properties in the treatment of diarrhea and dysentery. It also has hypoglycemiant, antioxidant, hepatoprotective, anti-allergic, antimicrobial, antigenotoxic, antispasmodic and anti-inflammatory action [1]. In the light of so many activities, it is suggested that this plant can help in controlling health-related infection, especially in areas of high microbiological risk, such as in the hospital environment. This work's objective, therefore, was to evaluate the antimicrobial activity of the *Psidium guajava*.

## Methods

An *in vitro *experimental study which evaluated the antimicrobial activity of leaves of the species *Psidium guajava*. After being dried at room temperature and ground, the vegetable material was extracted in ethanol (70%) at the Natural Resources Research Laboratory of the Federal University of Alagoas (UFAL), and the evaluation of the antimicrobial potential was undertaken at UFAL's Laboratory for Research in Wound Treatment. The antimicrobial activity was determined by microbial sensitivity tests, with the Agar perforation test and microdilution in broth for determining the Minimum Inhibitory Concentration (MIC). Nine lines of microorganisms were evaluated, including the Gram positive bacterias: *Staphylococcus aureus, Staphylococcus epidermides, Streptococcus pneumoniae*; Gram negative: *Pseudomonas aeruginosa, Enterobacter aerogenes, Escherichia coli, Acinetobacter calcoaceticus, Salmonella entérica*; and the fungus *Candida albicans*, all distributed by the American Type Cell Collection (ATCC). For the analysis of the Agar perforation test, two mehodologies were used [2,3]. The statistical analysis was undertaken using the ANOVA method, considered significant when p < 0.5.

## Results and conclusions

In the Agar perforation test, the sample demonstrated antimicrobial activity against the lines *S. aureus *(Inhibition zone: 12mm) and *P. aeruginosa *(Inhibition zone: 09 mm), evidencing a moderately active potential for the two methodologies, with bacterial inhibition of 42.85% and 32.72%, respectively, when compared with the inhibition of the positive standard control, ciprofloxacin (Inhibition zone: 28mm and 27.5mm respectively). The results obtained with the determination of the MIC demonstrated that the ethanolic extract of the leaf of *Psidium guajava *presented the lowest inhibitory concentrations, between 3.125 and 6.250 μg/mL, against the lines of *S. aureus, S. peumoniae, A. calcoaceticus *and *Candida albicans*. These are the first reports of the moderate antimicrobial activity of this part of the plant against the lines which already have strains which are resistant to the currently-used substances. In the light of this, it is important to invest in a phytochemical study of this raw extract, so as to isolate and identify its secondary metabolites, the aim being to strengthen this action. This evaluation could predict the chemical constituent(s) responsible for the biological activity [4], thus promoting the development of new alternatives in the control of infection, and the development of a product which could help in antimicrobial treatment based on a natural product.
